# A novel rat model of pulmonary artery embolism and the application value of lung ultrasound B‐lines in early‐stage pulmonary embolism

**DOI:** 10.1002/ame2.70250

**Published:** 2026-07-06

**Authors:** Huide Ma, Shuangrui Wang, Honghui Li, Yaxing Zhang, Yuman Gao, Jie Zhou, Xia Xu, Rong Wu

**Affiliations:** ^1^ Ordos Clinical Medical School of Inner Mongolia Medical University Ordos Inner Mongolia China; ^2^ Baotou Medical College Baotou Inner Mongolia China

**Keywords:** B line, Pulmonary embolism, Thoracic ultrasonography

## Abstract

**Background:**

Thoracic ultrasonography (TUS) is increasingly valued in the diagnosis of pulmonary thromboembolism (PE). Currently, clinical research using lung ultrasound relies solely on imaging methods and lacks histological evidence.

**Methods:**

A new rat pulmonary artery embolism model was prepared by injecting stable emboli through a visualized injection path. The effectiveness of the model was evaluated by assessing pulmonary artery hemodynamics, and the relationship between lung ultrasound and infarcted tissue was studied.

**Results:**

The new rat pulmonary emboli are stable, and ultrasound can display the distribution of the emboli in the pulmonary artery. Pulmonary artery hemodynamics can be used to evaluate the effectiveness of the model. The ultrasound manifestations of small patchy hemorrhagic infarcts on the surface of lung tissue after PE are B‐lines. Some small patchy hemorrhagic infarct lesions may progress to large infarct lesions, which are manifested as consolidation on ultrasound. The sensitivity of ultrasound B‐lines for detecting infarct lesions is 60.3%, and the specificity is 86.8%. The consistency boundary between the long diameter of pulmonary ultrasound B‐lines and the long diameter of pulmonary tissue infarction ranges from −0.75 to 2.05.

**Conclusions:**

This study demonstrates that ultrasound B‐lines have application value in the diagnosis of pulmonary embolism, and also provides a new animal model for research on pulmonary hypertension and the development of drugs for pulmonary embolism.

## Graphical abstract

We report a novel embolus‐induced PE rat model prepared via the inferior vena cava under ultrasound guidance. The embolus employed featured a tubular plastic casing filled with thrombus. By minimizing the contact surface between plasmin and thrombus, it effectively restrains the elevated plasmin activity in rats. This embolus may offer novel insights for establishing a reliable chronic thromboembolic pulmonary hypertension model. Additionally, both ends of the embolus can be exposed to either plasmin or thrombolytic agents, facilitating the evaluation of thrombolytic efficacy. Our emboli can intuitively show the distribution of emboli in the pulmonary arterial tree. The embolus is retrievable, and the efficacy of thrombolytic agents can be assessed by the plug recanalisation rate.

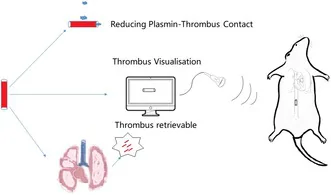

## 1 INTRODUCTION

Pulmonary thromboembolism (PE) is often caused by the detachment of lower extremity venous thrombi.^1^ The clinical manifestations of PE may be asymptomatic or may present with mild symptoms,^2^ and can even pose a threat to the patient's life.^3^ Early diagnosis and identification of PE are of great significance for risk stratification of PE,^4^, ^5^ and the treatment, follow‐up, and management of complications of PE are as significant as the treatment of PE itself.^6^ The diagnosis of PE requires computed tomography pulmonary angiography (CTPA),^7^ but CTPA cannot provide diagnostic evidence for patients with adverse reactions to contrast agents or pregnant women,^8^ nor can it offer bedside services, and the treatment cost is also high.^8^ In recent years, thoracic ultrasonography (TUS) has been increasingly valued in the diagnosis of pulmonary embolism (PE) due to its ability to be rapidly deployed in emergency settings, making it particularly suitable for critically ill patients or those who cannot tolerate CTPA.^9^, ^10^ Although the sensitivity of TUS alone for diagnosing PE is relatively low, a diagnostic strategy combining TUS with lower extremity deep vein ultrasound, right heart echocardiography, D‐dimer testing, and a Wells score >4 achieves both sensitivity and specificity exceeding 80%.^11^, ^12^ Currently, most TUS‐based pulmonary embolism (PE) studies rely solely on imaging evidence, and lack correlated histopathological evidence—particularly in patients with mild symptoms. Further research is needed to characterize post‐PE lung ultrasound findings and their correlation with histopathological changes in this population.

Animal models are needed to establish the correlation between lung ultrasound findings and PE‐induced pathological changes in lung tissue. The anatomical structure of the heart and lungs in rats, as well as their pathological features after embolism, are similar to those in humans, leading to their common use in research on pulmonary embolism (PE).^13^ Currently, there are several methods for establishing the PE (pulmonary embolism) model in rats, including the injection‐induced method using thrombi (autologous or allogeneic thrombus emboli),^14^, ^15^ the embolization method with non‐thrombotic materials,^16^, ^17^ and the surgical method.^18^, ^19^ The autologous thrombus injection method is suitable for studying the pathophysiological mechanisms of pulmonary embolism, but due to the high plasmin activity in rats, the thrombus self‐dissolution rate is high, leading to an unstable model.^20^ Non‐thrombotic material injection method can simulate complications such as pulmonary embolism and post‐embolism pulmonary hypertension, and is not suitable for the study of thrombolytic drugs.^21^ Direct surgical embolization requires mastery of surgical techniques and carries a high procedural mortality rate.^18^

This study establishes a stable rat model of PE by precisely injecting thromboemboli coated with polytetrafluoroethylene microtubes into the inferior vena cava under ultrasound guidance. It further investigates the early pulmonary ultrasound features of PE and their potential clinical applications with particular focus on the relationship between B‐lines and lung tissue alterations in the acute phase of pulmonary artery occlusion. The relationship between B‐lines on lung ultrasound and pulmonary consolidation was also observed.

## 2 METHODS

This study was approved by the Ethics Committee of Ordos Central Hospital (Ethics Approval No.: 2025‐233). A total of 28 healthy male SD rats, obtained from Beijing Vital River Laboratory Animal Technology Co., Ltd., with body weights of 400–500 g, were used in this study. Animals had ad libitum access to food and water and were housed at a controlled temperature of 25 ± 2°C.

### 2.1 Preparation of emboli and in vitro comparative experiments

The rats were anesthetized intraperitoneally with 1% pentobarbital sodium (40 mg/kg) (Sigma, St Louis, MO, USA) and the body temperature was maintained at 37 ± 0.5°C. After disinfecting the rat's tail vein, blood was collected via puncture and introduced into polytetrafluoroethylene microtubes (inner diameter = 0.3 mm, outer diameter = 0.6 mm) to induce polytetrafluoroethylene microtube thrombus (PTMT) formation. Similarly, blood was collected via tail vein puncture into a regular plastic tube in a homemade thrombosis generator (inner diameter = 0.6 mm) and left to stand for 30 min until the blood fully coagulated. Then the formed thrombus column was flushed into a Petri dish containing normal saline and rinsed three times with normal saline. The Petri dishes containing thrombus columns and the polytetrafluoroethylene thrombus columns were placed in a 4°C refrigerator and stored for 24 h. Then the thrombus columns were cut into pieces approximately 3–4 mm long using fine scissors, placed into two Petri dishes at room temperature and left to stand for 6 days, after which the morphological changes in the thrombus were observed.

### 2.2 Grouping and administration

Twenty‐eight SD rats were randomly divided into three groups: a normal control group, a sham operation group, and a PTMT group, with 7, 7, and 14 rats in each group, respectively. The normal control group received no treatment. The sham operation group rats were injected with 2 mL of normal saline via the inferior vena cava under ultrasound guidance. The PTMT group rats were injected with 1 mL of PTMT suspension plus 1 mL of normal saline via the inferior vena cava under ultrasound guidance to induce PE.

Rats in the normal control group, sham operation group, and PTMT group were evaluated for right ventricular structure and function via transthoracic echocardiography 1 h after modeling. Lung ultrasound assessments were performed at 1 week or 3 weeks post‐modeling in the PTMT group (Figure 1).

**FIGURE 1:**
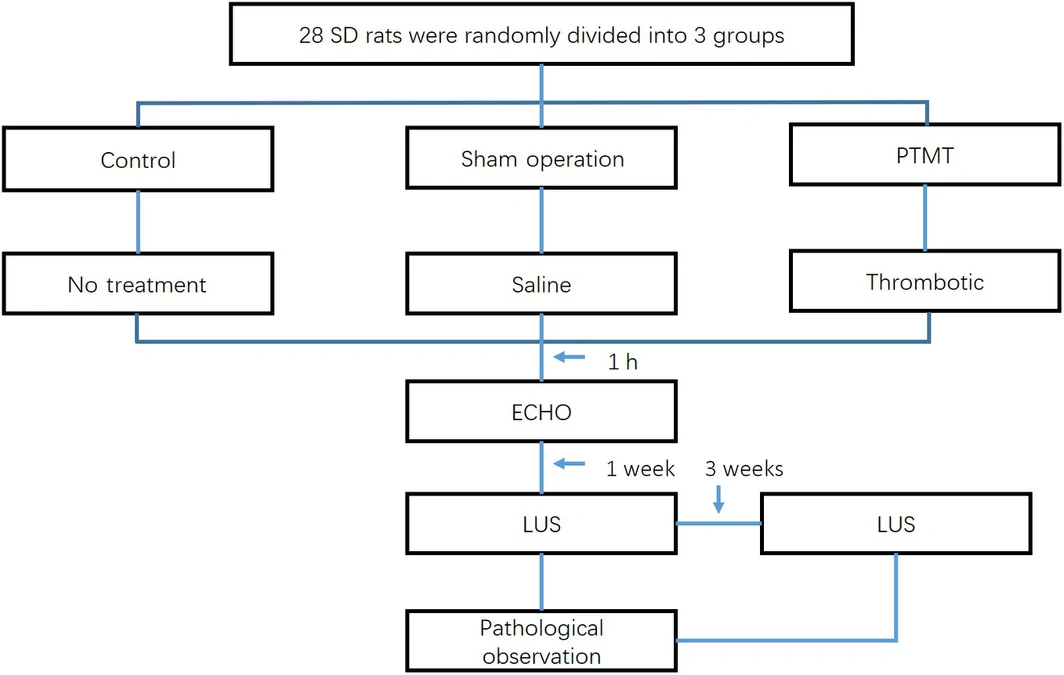
Flowchart of grouping and administration of rats in each group. ECHO, echocardiography; PE, pulmonary embolism; LUS, lung ultrasound.

### 2.3 Preparation of rat pulmonary embolism model and evaluation of model validity

Thirty PTMTs were prepared according to the previously described method. The PTMTs were placed in 18G indwelling needles connected to 5 mL syringes. After anesthetic treatment, the abdominal and thoracic hair of the rats was shaved, and the rats were fixed in a supine position on a surgical table and the abdominal skin was disinfected. Hold the linear array probe (Aloka Prosound F75, ALOKA, Japan) was held in the left hand, positioning it perpendicular to the abdominal skin. The location of the inferior vena cava and the puncture angle was identified, then under ultrasound guidance, an 18G indwelling needle was inserted into the rat's inferior vena cava (taking care to avoid the intestines) (Figure 3A). After observing the needle tip entering the inferior vena cava on the ultrasound image, the needle core was withdrawn (Figure 3B). A catheter was placed within the inferior vena cava, and blood return was observed. After confirming blood return, 1 mL of normal saline was injected to deliver the PTMT suspension. After the emboli injections were completed, the catheter needle was withdrawn and manual compression was applied to the abdominal puncture site for 10 min. The animal was placed in a thermostatically controlled recovery chamber. One hour later, the right ventricular outflow tract diameter (RVOTD), pulmonary ejection time (ET), and pulmonary artery acceleration time (PAAT) were measured and recorded. The rats were then returned to their cages for continued breeding and observation.

### 2.4 Lung ultrasound evaluation of rat pulmonary embolism model

One week or three weeks after modeling, lung ultrasound was used to observe B‐lines and consolidation in the lungs. A linear probe was used for transverse scanning from the head side to the tail side of each intercostal space. Each intercostal space was divided into several equal sections of approximately 4 mm along the costal margin from the sternal end to the vertebral end of the ribs. The position and long diameter of B‐lines or consolidation along the long axis of the costal margin were measured and recorded. Using our method for contrast‐enhanced ultrasound of the consolidated lung tissue in an animal model, the ultrasound probe should be fixed, and 0.5 mL of contrast agent should be injected via the tail vein. Contrast‐enhanced ultrasound imaging should be performed on the Mindray A20 ultrasound system (Mindray, China).

### 2.5 Lung histological evaluation of rat pulmonary embolism model

After the ultrasound examination was completed, the animals were euthanized with an overdose of isoflurane. Large scissors were used to cut the ribs and open the thoracic cavity, taking care to avoid damaging the lungs and major blood vessels. The needle of the infusion device was inserted into the left ventricle and into the aorta. The inferior vena cava and the left atrium were cut with scissors. Heparin normal saline (heparin sodium: normal saline = 25 000 U: 250 mL) was infused, at a controlled perfusion rate of 50 mL per minute. After the heparinized normal saline perfusion was completed, the needle of the infusion device was inserted into the right ventricle and through to the pulmonary artery, and 80 mL of 4% paraformaldehyde was infused to fix the lung tissue. After fixation was completed, the lungs, heart, trachea, esophagus, thymus, and major blood vessels were separated from the thoracic cavity as a whole. The esophagus, thymus, and surrounding adipose tissue were carefully removed, leaving only the heart, lungs, and major blood vessels. The location and length of the pulmonary surface infarction were recorded and measured. The lung tissue was then placed in 4% formaldehyde solution and left to stand for 24 h. Subsequently, the tissue was transferred to physiological saline, and an ultrasound diagnostic instrument was used to locate emboli (Figure 3C). The lung tissue was sectioned into uniform slices of equal width, and emboli were manually isolated. The extent of thrombus lysis within each embolus was quantified and recorded. Another rat was anesthetized and perfused, and then dissected. Lung cross‐sections were prepared using the aforementioned method and fixed in 10% formalin buffer solution, followed by paraffin embedding. The sections were stained with hematoxylin and eosin (H&E) for histopathological evaluation.

### 2.6 Statistical analysis

All data were analyzed using GraphPad Prism 10, Microsoft Excel 2016, and ImageJ software. To evaluate the effectiveness of the model, one‐way analysis of variance (ANOVA) with Tukey's post hoc test was used to compare RVOTD, PAAT, and ET between the model group and the control group. To evaluate the diagnostic performance of lung ultrasound B‐lines in a pulmonary embolism model, we used the infarct in the corresponding intercostal space of the rat lung tissue as the standard, and calculated the sensitivity and specificity between lung ultrasound B‐lines and gross specimen infarcts. We used the Bland–Altman plot method to verify the consistency between the long‐axis measurements of B‐lines by lung ultrasound and those of pulmonary infarcts measured on gross specimens. To indicate the stability of the emboli, the recanalization rate of the emboli was used: Recanalization rate = Number of recanalized emboli recovered by the tissue / Total number of emboli recovered by the tissue.

## 3 RESULTS

### 3.1 Stability of PTMT in vitro

Figure 2A,B shows the morphological changes of the PTMT and autologous thrombus at 1, 3, and 6 days, both in whole and segmented emboli. The PTMT on days 1, 3, and 6 appears bright red, with no significant changes in thrombus color over time. The overall morphology remains intact, with smooth edges, and no obvious fragmentation or dissolution is observed. The autologous thrombus appears dark red, with the color gradually lightening over time. The structure is loose, exhibiting prominent rough edges and a small amount of debris, while partial thrombus dissolution is observed.

**FIGURE 2:**
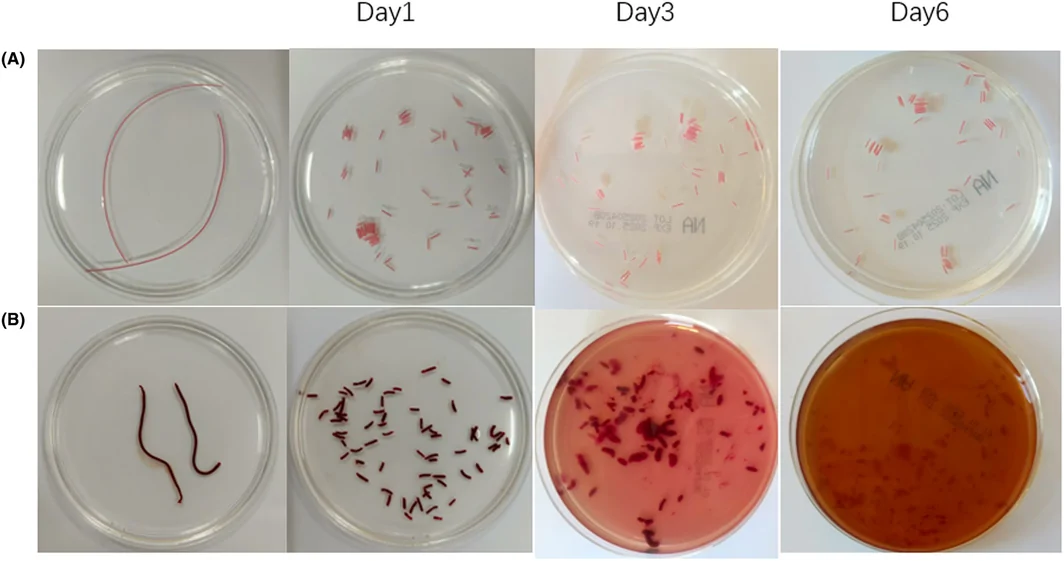
Stability of emboli. Morphological changes of PTMT columns (A) and autologous thrombus columns (B) over time.

### 3.2 Preparation and validation of rat pulmonary embolism model

Under ultrasound guidance, an 18G indwelling needle loaded with emboli was inserted into the inferior vena cava of rat, and the emboli were slowly infused into the inferior vena cava with normal saline (Figure 3A,B). The B‐mode ultrasound image of the lung tissue of the model rat (Figure 3C) shows an equal sign‐like strong echo embolus located within the lung tissue. The lung tissue identified by the ultrasound localization was incised, and the embolus was found to be located in the pulmonary artery, as shown in Figure 3D.

**FIGURE 3:**
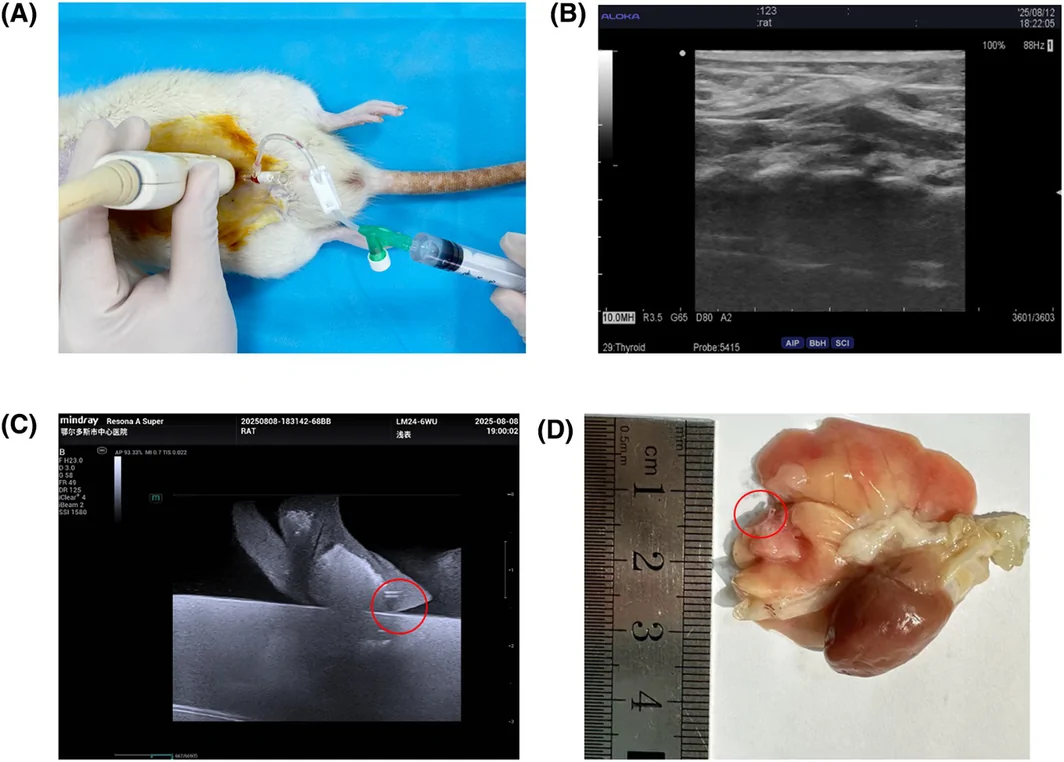
Preparation and validation of rat pulmonary embolism model. (A) Under ultrasound guidance, an 18G indwelling needle loaded with emboli was inserted into the inferior vena cava of rat. (B) B‐mode ultrasound image showing an 18G catheter sheath positioned within the inferior vena cava. (C) In the ultrasound B‐mode image of the rat lung tissue, the PTMT echo is visible (red circle). (D) Lung tissue with PTMT (red circle) is visible within the pulmonary artery.

### 3.3 Validation of the in vivo rat pulmonary embolism model

The short‐axis parasternal echocardiographic view of the rat heart visualizes the pulmonary artery and its Doppler flow signal, allowing simultaneous measurement of RVOTD (white double arrow, Figure 4A,A′). Spectral Doppler measures the PAAT and ET of the pulmonary artery (Figure 4B,C), and the above measurement results are presented in Table 1. There was no statistically significant difference in RVOTD and hemodynamic parameters between the normal control group and the sham surgery group. Compared with the above groups, the PTMT group rats showed increased RVOTD (RVOTD: 3.63 ± 0.16 vs. 2.58 ± 0.17/2.62 ± 0.12, *p* < 0.0001), shortened PAAT (PAAT: 26.00 ± 3.41 vs. 32.83 ± 4.07/34.83 ± 2.14, *p* < 0.01), and decreased PAAT/ET ratio (0.23 ± 0.04 vs. 0.41 ± 0.05/0.46 ± 0.03, *p* < 0.0001) (Figure 5A–C).

**FIGURE 4:**
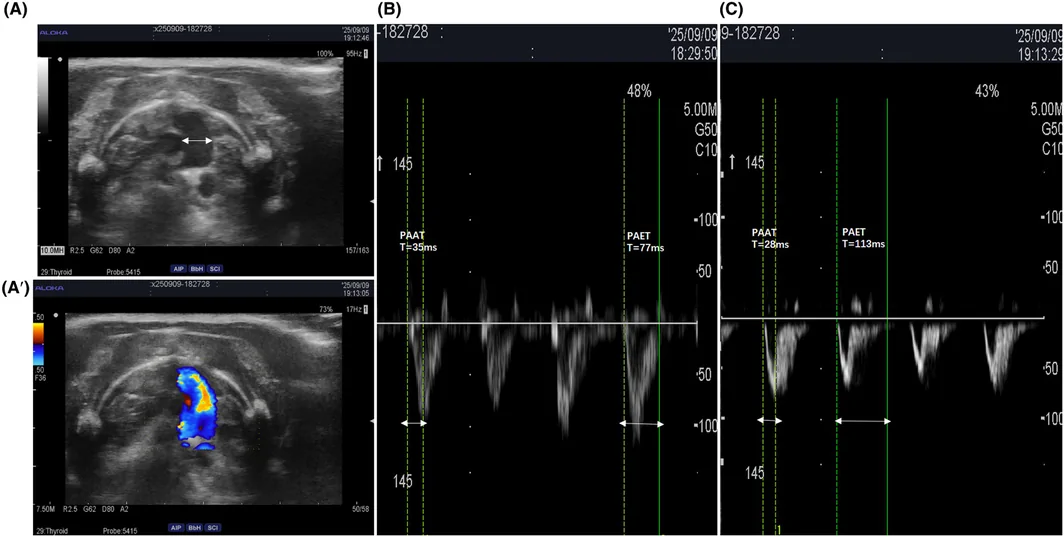
PA hemodynamic parameters measured. (A, A′) RVOTD measurement (white double arrow) and pulmonary artery blood flow imaging. (B, C) Spectral Doppler measures the PAAT and ET (white double arrow).

**TABLE 1:** PA hemodynamic parameters measured.

Group	*N*	RVOTD (mm)	PAAT (ms)	PAAT/ET
Normal control	6	2.62 ± 0.12	34.83 ± 2.14	0.46 ± 0.03
Sham operation	6	2.58 ± 0.17	32.83 ± 4.07	0.41 ± 0.05
PTMT	12	3.63 ± 0.15	26.00 ± 2.80	0.23 ± 0.03

*Note*: Mean ± SD. 1 h after injection of saline/PTMT, rats' PA morphology and function in normal control, sham operation and PTMT groups were evaluated by ECHO. *p*‐Values from One‐Way ANOVA.

Abbreviations: ET, pulmonary ejection time; PATT: pulmonary artery acceleration time; RVOTD, right ventricular outflow tract diameter.

**FIGURE 5:**
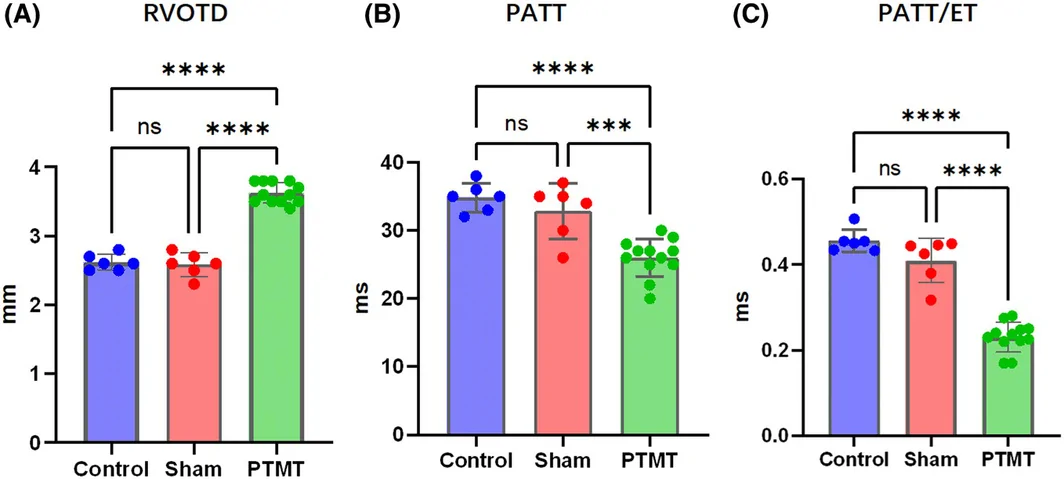
ROVTD PATT and ET in all groups. 1 h after injection of saline/PTMT, rats' RVOTD, PATT and ET in normal control (*n* = 6), sham operation (*n* = 6) and PTMT (*n* = 12) groups were evaluated by ECHO. Rats in PTMT group (A) had larger RVOTD and lower PATT, PATT/ET than in normal control (B) or sham operation (C) groups. ET, Pulmonary ejection time; PATT, pulmonary artery acceleration time; RVOTD, right ventricular outflow tract diameter. The value bars with ns are not significant, asterisk denotes statisically significant differences *** *p* < 0.001; **** *p* < 0.0001.

### 3.4 Lung ultrasound evaluation of rat pulmonary embolism model

Lung ultrasound in rats at 1 week post‐induction revealed B‐lines (Figure 6A, red arrow), while rat at 3 weeks showed lung consolidation (indicated by red circle in Figure 6B). Sonographic contrast imaging revealed no significant enhancement within the consolidated region (white circle, Figure 6C). Gross examination of lung specimens from a rat at 1 week post‐induction demonstrated multiple pinpoint hemorrhagic infarcts on the pleural surface (red circle, Figure 6D). Figure 6E is a schematic diagram illustrating the measurement of B‐lines using lung ultrasound and the quantification of discrete pulmonary infarcts in rat at 1 week post‐pulmonary embolism induction. This indicates that B‐lines correspond spatially to microinfarct foci on the lung surface. Figure 6F shows lung tissue from a rat at 3 weeks post‐induction, revealing pinpoint hemorrhagic infarcts that have formed a large confluent infarct on the pleural surface (red circle). Figure 6G is a diagram illustrating the quantitative recording method for B‐lines and infarct areas in the lungs of model rat one week later. The red circle indicates the measurement value of B‐lines, and the black circle indicates the measurement value of pulmonary surface infarction. Table 2 shows the number of B‐line positives and negatives detected by lung ultrasound in 240 equal regions (*n* = 6) in the 1‐week model rat, as well as the number of positives and negatives for the presence of small patchy hemorrhagic infarct foci on the surface of lung tissue. Taking the appearance of hemorrhagic infarction foci on the surface of lung tissue as the standard, the sensitivity of ultrasound B‐lines in detecting infarction foci is 60.3%, and the specificity is 86.8%. Figure 6H shows that in the Bland–Altman plot analysis of 76 regions where both ultrasound B‐lines and pulmonary surface infarcts were positive, the limits of agreement between the long diameter of B‐lines detected by lung ultrasound and the long diameter of pulmonary infarcts on the tissue surface were −0.75 to 2.05, with most data points falling within the limits of agreement (Figure 6H).

**FIGURE 6:**
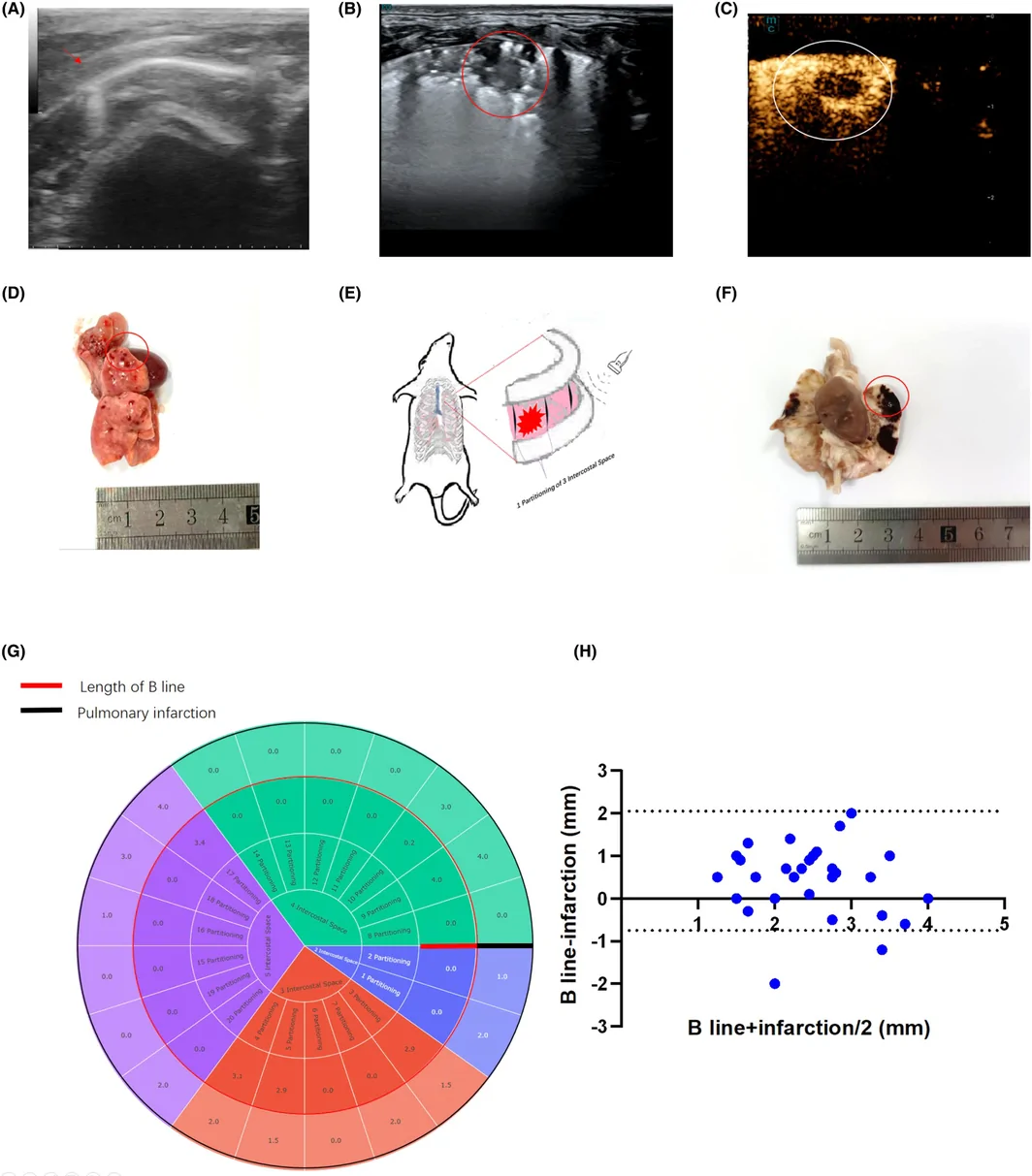
Lung ultrasound evaluation of rat pulmonary embolism model. (A) Lung ultrasound in rat at 1 week post‐induction revealed B‐lines (red arrows). (B) Lung ultrasound in rat at 3 week post‐induction revealed consolidation (red circle). (C) Sonographic contrast imaging revealed no significant enhancement within the consolidated region (white circle). (D) Rat at 1 week post‐induction demonstrated multiple pinpoint hemorrhagic infarcts on the pleural surface (red circle). (E) Schematic diagram illustrating the measurement of B‐lines using lung ultrasound and the quantification of discrete pulmonary infarcts in rats at 1 week post‐pulmonary embolism induction. (F) Three weeks later, the model's lung tissue showed large areas of infarction on the lung surface (as indicated by the red circles). (G) A diagram illustrating the quantitative recording method for B‐lines and infarct areas in the lungs of model rat one week later. The red circle indicates the measurement value of B‐lines, and the black circle indicates the measurement value of pulmonary surface infarction. (H) Mean ± SD of long axis length of B‐lines and pulmonary infarcts in rats at 1 week post‐induction.

**TABLE 2:** Ultrasound B‐lines (+/−) and pulmonary infarction (+/−).

Pulmonary infarction				
		+	−	Total
**B line**	+	76	15	91
	**−**	50	99	149
**Total**		126	114	

### 3.5 Embolic stability evaluation

In the 3‐week model rat, there were large, deep red infarction areas around the lung surface (Figure 6F). A regular, circular, and transparent PTMT was observed within the pulmonary artery on HE staining (Figure 7A). The thrombus fills the lumen of the ring‐like embolus (as shown in the enlarged view, Figure 7B). Numerous red blood cells were observed in the alveolar spaces (black arrow, Figure 7A). After isolation from the lung tissue, most emboli did not recanalize, resulting in an embolus recanalization rate of approximately 6%–13%.

**FIGURE 7:**
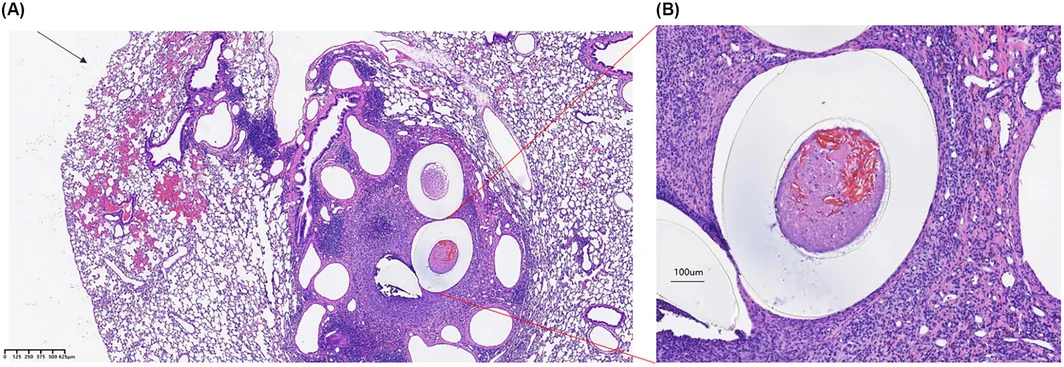
Histological evaluation (H&E) of lung tissue in the 3 week model rat. (A) Three weeks after modeling, there are obvious areas in the rat lung tissue indicating hemorrhagic infarction caused by embolism (black arrow). (B) Within the pulmonary artery, there is a regular round‐shaped transparent PTMT embolus (enlarged view), with thrombus filling the ring‐like embolus.

## 4 DISCUSSION

In current rat PE models, it is very difficult to simultaneously ensure embolus stability and conduct application research in drug development.^13^ We report a novel embolus‐induced PE rat model prepared via the inferior vena cava under ultrasound guidance. The embolus employed in this model offers the following advantages: (1) Given the high plasmin activity in rats,^22^ autologous thrombus is not suitable for establishing a model of chronic thromboembolic pulmonary hypertension. The embolus employed in our model featured a tubular plastic casing filled with thrombus. This design combines the advantages of both plastic and thrombus emboli. By minimizing the contact surface between plasmin and thrombus, it effectively restrains the elevated plasmin activity in rats. This embolus may offer novel insights for establishing a reliable chronic thromboembolic pulmonary hypertension model. Additionally, both ends of the embolus can be exposed to either plasmin or thrombolytic agents, facilitating the evaluation of thrombolytic efficacy (Figure 2). (2) Currently, visualization of thrombi within pulmonary arteries typically requires a pulmonary artery cast, a method that is operationally complex and costly. Furthermore, excessive injection of the casting medium may lead to the formation of a pulmonary vein cast, thereby impairing the visualization of emboli within the pulmonary arterial tree. Since emboli are echogenic under ultrasound, artificial pulmonary consolidation was induced in this experiment by evacuating air from the lungs. This method can intuitively show the distribution of emboli in the pulmonary arterial tree, and it is simple to operate at a low cost (Figure 3 C, D). (3) The embolus is retrievable, and the efficacy of thrombolytic agents can be assessed from the plug recanalisation rate (Figure 7).

Typically, the path of embolus injection involves external jugular veins and tail veins.^23^, ^24^ Procedures through these routes make it difficult to visualize the above path operations, and catheterization often leads to venous occlusion. In this model, we selected ultrasound‐guided embolus delivery via the inferior vena cava, where ultrasound can visually display the catheter placed in the inferior vena cava. This approach is minimally invasive and reduces the risk of venous thrombosis (Figure 3A,B).

Typically, the B‐lines generated by pulmonary embolism (PE) cannot be distinguished from those caused by other lung diseases that lead to reduced alveolar gas and increased interstitial edema. Therefore, the ultrasound diagnosis of PE primarily relies on the combination of transthoracic ultrasound (TUS), peripheral venous ultrasound, echocardiography, and multi‐organ ultrasound.^11^ Data indicate that multiple assessments, including ultrasound B‐lines, lower extremity venous thrombosis and right ventricular overload, and clinical risk stratification, can improve the accuracy of PE diagnosis and serve as effective alternatives when CTPA and angiography are not feasible to confirm PE.^12^ The diagnosis of PE by ultrasound is analogous to the validation of model effectiveness in this experiment. Additionally, the validation of model effectiveness is also a prerequisite for the application of animal models. The validity of the model can be preliminarily judged by evaluating the pulmonary hemodynamics. Pulmonary hemodynamics can be obtained through right heart catheterization and echocardiography.^25^ However, right heart catheterization is complex, invasive, and associated with adverse reactions, which limits its application.^26^ Echocardiography is non‐invasive and simple to perform. The parameters obtained, such as PAAT and PAAT/ET, can reflect pulmonary artery pressure and right heart load.^27^ This study employed echocardiography to measure pulmonary artery hemodynamics after modeling (Figure 4). Compared with the control group, the pulmonary artery diameter was widened, PAAT time was shortened, and PAAT/ET ratio was reduced, which were consistent with the ultrasonographic findings of pulmonary artery pressure elevation caused by pulmonary embolism. The successful preparation of the model was confirmed by echocardiography, which provided a basis for further experiments.

The lungs are supplied with blood by the pulmonary artery and bronchial artery. After pulmonary embolism occurs, some lung tissue will progress to infarction, and this process is progressive.^28^ To simulate this process, we coated the thrombus with polytetrafluoroethylene, which effectively inhibited the hyperfibrinolytic activity in rats. This allowed the thrombus to remain in the pulmonary artery for a longer duration, resulting in the formation of a stable pulmonary arterial embolism. Pulmonary artery embolism can lead to alveolar hemorrhage and necrosis.^29^ Due to reduced secretion of pulmonary surfactant, atelectasis occurs. Increased secretion of inflammatory mediators enhances vascular permeability, resulting in alveolar edema. The above pathophysiological processes typically occur during the acute phase of pulmonary artery embolism.^9^, ^30^ Alveolar air reduction and interstitial edema are manifested on ultrasound as hyperechoic artifacts perpendicular to the chest wall, known as the B line.^31^ This study applied lung ultrasound to observe that pulmonary B‐lines appear after modeling, typically within 1 week post‐modeling (Figure 6A). Using intercostal zone localization (Figure 6E), we observed that in model rats, the areas where lung ultrasound showed B‐lines were histologically confirmed to be small patchy hemorrhagic infarctions (Figure 6D). In rats 1 week after modeling, lung tissue surface with small patchy infarct lesions was the standard, and the number of infarct lesions detectable by lung ultrasound B‐lines in 240 equally divided regions was counted. The sensitivity of ultrasound B‐lines was calculated to be 60.3%, and the specificity was 86.8%. The results indicate that ultrasound B‐lines are a specific manifestation of small patchy hemorrhagic infarction in lung tissue and therefore have significant value in multi‐organ ultrasound for the diagnosis of pulmonary embolism. In 76 lung regions confirmed as infarcts by both lung ultrasound B‐lines and lung histology, we found a good consistency between the measured values of lung B‐lines and the measured values of tissue infarcts, The B‐line measurements were slightly higher than the tissue infarction measurements, and we speculate that this is due to the alveolar gas in the free lung tissue being expelled, leading to a reduction in lung tissue volume. As a result, the long diameter of the measured infarcted area is smaller than the long diameter of the lung ultrasound B‐lines. Therefore, the ultrasound B‐line measurement of the infarcted area range is closer to the range of lung tissue involvement under in vivo conditions. Experimental results indicate that ultrasound B‐lines can be used for quantitative analysis of the size of pulmonary infarct lesions during the acute phase of pulmonary embolism, and can serve as a tool for evaluating the efficacy of PE treatment.

If the alveolar air further decreases and the interstitial edema further worsens, it will present as solid parenchyma on ultrasound, termed consolidation.^32^, ^33^ In this study, lung consolidation usually occurred 3 weeks after modeling (Figure 6B,C). The lung ultrasound of the 3 week rat after modeling showed consolidations (Figure 6B,C), which were histologically confirmed as a confluent small patch‐like hemorrhagic infarction (Figure 6F). Contrast‐enhanced ultrasound can also provide evidence of pulmonary consolidation as infarction foci in lung tissue, with no blood perfusion in the consolidation area (Figure 6C). Our experimental results show that after PE occurs, pulmonary infarction is prone to appear near the pleura. In the early stage, it is a small focused area of infarction, and then it progresses to a large area of infarction. This is consistent with the concept that pulmonary infarction is prone to occur near the pleura.^34^, ^35^, ^36^ Early pulmonary ultrasound findings in PE are B‐lines. B‐lines can progress to consolidation. B‐lines represent small areas of infarction, while consolidation indicates the fusion of these small infarcted areas into larger infarcted regions. B‐lines are the ultrasound manifestation of early pulmonary infarction in PE, while consolidation represents the ultrasound appearance of end‐stage pulmonary tissue infarction due to PE. The progression from ultrasound B‐lines to pulmonary consolidation is a gradual process. Lung ultrasound can depict the pathophysiological process of pulmonary embolism, providing imaging evidence for the clinical staging of PE.

The limitations of this study include: (1) Pulmonary ultrasound can only display pulmonary tissue in the intercostal spaces due to rib obstruction, and cannot show the mediastinal surface of the lung nor pulmonary tissue obstructed by ribs. (2) When the infarct foci in pulmonary tissue are small with slight bleeding, they are difficult to identify as infarct foci during histological measurement, which may lead to a reduced specificity of ultrasound B‐lines.

In conclusion, we have successfully established an improved rat PE model characterized by an ultrasound‐guided minimally invasive approach and stable and recyclable emboli. This model provides research tools for the development of PE drugs and the prevention and treatment of complications. Pulmonary ultrasound B‐lines have potential value in the diagnosis of pulmonary infarction, especially considering the high specificity of infarction foci within the intercostal spaces.

## AUTHOR CONTRIBUTIONS

**Huide Ma:** Data curation; formal analysis; methodology; project administration; supervision; writing – original draft; writing – review and editing. **Shuangrui Wang:** Methodology. **Honghui Li:** Data curation; methodology. **Yaxing Zhang:** Formal analysis; investigation. **Yuman Gao:** Investigation; methodology. **Jie Zhou:** Methodology. **Xia Xu:** Supervision; writing – review and editing. **Rong Wu:** Funding acquisition; resources; supervision; writing – review and editing.

## FUNDING INFORMATION

This research was supported by the Inner Mongolia Autonomous Region Natural Science Foundation (No. 2025MS08053), Collaborative Research Project for Public Hospitals (No. 2024GLLH1307).

## CONFLICT OF INTEREST STATEMENT

The authors declare no competing interests.

## ETHICS STATEMENT

This study was approved by the Ethics Committee of Ordos Central Hospital (approval no.: 2025‐233).
